# The reproductive tract microbiota in pregnancy

**DOI:** 10.1042/BSR20203908

**Published:** 2021-09-06

**Authors:** Karen Grewal, David A. MacIntyre, Phillip R. Bennett

**Affiliations:** 1 Tommy’s National Centre for Miscarriage Research, Hammersmith Hospital Campus, Du Cane Road, London, U.K.; 2 Institute of Reproductive and Developmental Biology, Imperial College London, Hammersmith Hospital Campus, Du Cane Road, London, U.K.; 3 March of Dimes European Prematurity Research Centre, Hammersmith Hospital Campus, Du Cane Road, London, U.K.

**Keywords:** Microbiota, Pregnancy, Reproduction

## Abstract

The reproductive tract microbiota plays a crucial role in maintenance of normal pregnancy and influences reproductive outcomes. Microbe–host interactions in pregnancy remain poorly understood and their role in shaping immune modulation is still being uncovered. In this review, we describe the composition of vaginal microbial communities in the reproductive tract and their association with reproductive outcomes. We also consider strategies for manipulating microbiota composition by using live biotherapeutics, selective eradication of pathogenic bacteria with antibiotics and vaginal microbiota transplantation. Finally, future developments in this field and the need for mechanistic studies to explore the functional significance of reproductive tract microbial communities are highlighted.

## Introduction

Infection has long been recognised as an important risk factor of poor reproductive success. In early pregnancy, infection is implicated in 15% of early and 66% of late miscarriage [1]. Towards the end of pregnancy, it is associated with approximately 40% of all preterm birth cases [2]. Like other body niches, the lower reproductive tract has co-evolved alongside a rich microbial community that has permitted the formation of important symbiotic relationships that play a crucial role in health and disease [3,4]. While clinical microbiology has enabled the identification of specific reproductive tract pathogens associated with increased risk of adverse pregnancy outcomes (e.g. *Chlamydia trachomatis* [5]), recent application of high-throughput bacterial DNA sequencing methods has deepened our understanding of how microbiota composition and host interactions effect pregnancy outcomes.

## Techniques used to characterise the reproductive tract microbiota

The 21^st^ century has seen a dramatic improvement in our ability to study the human microbiome because the limitations of culture and microscopy-based investigations have largely been superseded by molecular-based approaches, many of which are based upon high-throughput sequencing of bacterial DNA. Culture-based techniques, which have been used since the early 20^th^ century, are labour-intensive and provide a limited view of the diversity of bacteria in any particular body site. The great majority of bacteria present in the human body require very specific culture conditions which makes comprehensive analysis of bacterial communities by culture almost impossible. Although more sophisticated culture approaches using enhanced culture techniques and microbial culture chips have been developed, the growth of some organisms depends on the metabolic activity of others, which leads to a number of limitations to these techniques [6]. High-throughput DNA sequencing approaches have become increasingly affordable enabling their widespread use for characterisation of complex microbial communities and estimation of the relative abundances of microorganisms in a given body site. Two main sequencing strategies have emerged. Firstly, shotgun metagenomics involves sequencing whole community DNA (bacterial, viral, fungal and host). This technique has the advantage that it potentially explores the genetic diversity and function of the microbiota, and is not limited just to bacteria. A disadvantage is that under certain circumstances, a significant proportion of the DNA being sequenced is of host origin. A more widely used technique, commonly termed metataxonomics, metabarcoding or amplicon sequencing, focuses on sequencing and amplifying specific regions of the bacterial 16S ribosomal RNA gene (16S rRNA) [3,6]. This gene is present in all bacterial species in a variety of copy numbers. It consists of nine variable regions flanked by regions of greater homology across bacterial species. PCR primers can be designed to hybridise to the conserved regions and amplify across one or more variable regions. The identity of the microorganism at genus, species and sometimes strain level can be determined from the DNA sequence of the variable region [6,7].

## Metataxonomics-based characterisation of vaginal microbiota communities

It has been long established that the vagina is not a sterile environment. Gustav Doderlein first described Lactobacilli in 1892 and subsequent work has shown that Lactobacilli dominate most vaginal microbial communities [8]. There is a large body of evidence to suggest that microbial communities that colonise the vagina promote homoeostasis and have a substantial impact on reproductive health [9–11]. Taxonomic profiles of vaginal microbial communities can be sorted into a discrete number of categories based on hierarchical clustering of the pairwise distances between samples. This is advantageous because collapsing a hyperdimensional taxonomic profile into a single categorical variable facilitates data exploration, epidemiological studies and statistical modelling. One of the first studies to apply this approach to vaginal microbial communities was by Ravel and colleagues [12] who examined samples taken from 396 asymptomatic reproductively aged women. At species level, hierarchical clustering analysis characterised the vaginal microbiota into five community state types (CSTs), four of which were characterised by high relative abundance of specific *Lactobacillus* species [12]: CST I – *Lactobacillus crispatus (L. crispatus)*, CST II – *Lactobacillus gasseri (L. gasseri)*, CST III – *Lactobacillus iners* (*L. iners)*, CST IV – *‘high diversity’*, CST V – *Lactobacillus jensenii (L. jensenii).* The CST IV (high diversity) group was characterised by a low abundance of *Lactobacillus* spp. and an overrepresentation of anaerobic bacteria such as *Atopbium, Prevotella, Sneathia, Gardnerella* and *Mobiliuncus* [13]. A number of these taxa have been associated with bacterial vaginosis (BV), a polymicrobial disorder that is associated with preterm birth [14], higher risk of acquiring sexually transmitted infections [15] and late miscarriage [16–19].

Other studies using different patient populations have used various forms of clustering analysis to define vaginal microbiome groups or vagina community states specific to those individual patient populations. Recently Ravel and colleagues [20] sought to standardise and advance the assignment of samples to CSTs by creating VALENCIA (VAginaL community state typE Nearest Centroid clAssifier), a nearest centroid-based tool which classifies samples based on their similarity to a set of racially, ethnically and geographically diverse reference datasets. This approach allows any individual microbiota community to be assigned to 1 of 13 CSTs. There are six *Lactobacillus* spp. dominant CSTs, I-A, I-B, II, II-A, II-B and V which correspond to the original CST defined by Ravel and colleagues [12], but with the designation expanded to allow for community states that contain a combination of organisms. The original *Lactobacillus* spp. depleted CST IV is expanded in VALENCIA into CST IV-A, IV-B and five CST IV-C (0–4), to account for the variety of different *Lactobacillus* spp. deplete bacterial communities. *The main advantage of this classification system is that it can characterise the vaginal microbiome in a standardised way to allow comparison of different study datasets. There is a move away from individual classification systems that are not comparable and a drive towards laying different datasets on to this framework.*

## Vaginal microbiota and reproductive outcomes

The composition of the vaginal microbiota in pregnancy displays a higher abundance of *Lactobacillus* spp. and more stability throughout the entire pregnancy. In both pregnant and non-pregnant women, the vaginal microbiota can fluctuate and transition from one CST to another. There are a variety of factors, such as ethnicity, hygiene practises, hormonal fluctuation and contraceptives, that influence the structure and composition of the vaginal microbiota. One of the first longitudinal studies that characterised the vaginal microbiota using DNA sequencing techniques in pregnant and non-pregnant women found that high diversity communities were rarely seen in pregnant women who delivered at term [13]. Even though bacterial communities in pregnancy did appear to shift between CSTs dominant in *Lactobacillus* spp., they rarely transitioned to CST IV. *Lactobacillus* spp. stability in pregnancy may represent an evolutionary adaptation to enhance reproductive fitness and protect against ascending infection. The stability may also be driven by high oestrogen levels in pregnancy as the post-partum state is characterised by a dramatic shift to less *Lactobacillus* spp. dominant communities with increased α diversity [21].

### Assisted conception

Several studies have focused on how the vaginal microbiota influences assisted reproductive technology outcomes. In a prospective study of 130 infertility patients undergoing *in-vitro* fertilisation (IVF), the clinical pregnancy rate was significantly lower in women with an ‘abnormal’ microbiota (high concentrations of *Gardnerella vaginalis* and/or *Atopobium vaginae*) [22]. In a recent study the vaginal microbiota and metabolome was characterised in recurrent implantation failure (RIF) patients (*n*=27) compared with patients who achieved a clinical pregnancy with their first frozen embryo cycle (*n*=40). Vaginal microbiota dominant in *Lactobacillus* spp. was again related to clinical pregnancy while RIF patients had increased microbial diversity [23]. Other fertility studies have also shown that a *Lactobacillus* spp. dominant vaginal microbiota is associated with positive pregnancy outcomes. However, many of these studies have small samples sizes and heterogeneity in their patient populations [24,25].

### Miscarriage

There is currently a relative paucity of data exploring the pregnancy vaginal microbiota and adverse early pregnancy events. Recent work has shown reduced abundance in *Lactobacillus* spp. and increased α diversity with first trimester miscarriage [26,27]. *Lactobacillus*-depleted vaginal microbiota also appears to be a risk factor for ectopic pregnancy [28]. There is limited evidence investigating the early placental pregnancy microbiome and how that relates to reproductive outcome. One recent study used quantitative polymerase chain reaction (qPCR) to test for presence of *Mollicutes* in endocervical swabs and placental tissue collected early in pregnancy from women experiencing miscarriage (*n*=89) and controls (*n*=20). Detection of *Mollicutes* in the placenta was associated with miscarriage and there was also a significant increase in microbial load of *Mycoplasma hominis, Ureaplasma urealyticum* and *Ureaplasma parvum* in miscarriage patients compared with the control group. While the authors proposed that this represented ascending infection of placental tissue leading to the adverse event, the presence of these organisms in cervical swabs suggests possibility of contamination during sample collection [29].

Table 1 gives examples of studies which have explored the relationship between the vaginal microbiota and early pregnancy as well as assisted reproduction.

**Table 1:** Examples of studies which explore the relationship between the vaginal microbiota and early pregnancy and assisted reproduction

Author/year	Sample	Sample size	Population	Risk of adverse outcome		Findings
				High diversity	*L. crispatus/L. dominance*	
Early pregnancy and assisted conception						
Hyman et al./2012, *J. Assist. Reprod. Genet.* [24]	Vaginal swabs	30 women	IVF	Increased diversity in those who failed to achieve clinical pregnancy	No protective effect of *Lactobacillus* spp.	The vaginal microbiota on the day of embryo transfer affects pregnancy outcome.
Haahr et al./2016, *Hum. Reprod.* [22]	Vaginal swabs	130 women (qPCR-specific for *Lactobacillus* spp., *G. vaginalis*, *A. vaginae*)	Completed IVF treatment (*n*=84)			Significantly lower clinical pregnancy rate in those patients with abnormal vaginal microbiota
Haahr et al./2019, *J. Infect. Dis.* [99]	Vaginal swabs	120 women	IVF	Clinical pregnancy and live birth rate was less likely higher diversity		Overall there was no significant association between CST and reproductive outcome
Koedooder et al./2019, *Hum. Reprod.* [100]	Vaginal swabs	192 women	IVF (fresh embryo transfer)		Higher chance of pregnancy when dominated by *L. crispatus*	Women with a lower percentage of *Lactobacillus* spp. were less likely to have successful embryo implantation
Wee et al./2019, *Aust. N.Z. J. Obstet. Gynaecol.* [101]	Vaginal swabs Cervical swabs Endometrial sample	31 women Control (*n*=16) Cases (*n*=15)	History of infertility compared with those with history of fertility			A trend towards infertile women having more *Ureaplasma* in their vagina and *Gardnerella* in cervix
Al-Memar et al./2020, *BJOG* [26]	Vaginal swabs	Miscarriage (*n*=78) Term (*n*=83)	Early pregnancy	Increased risk first trimester miscarriage		First trimester miscarriage associated with reduced *Lactobacillus* spp. dominance and increased diversity and richness
Fu et al./2020, *mBio* [23]	Vaginal swabs	RIF (*n*=27) Control (*n*=40)	RIF and Control (clinical pregnancy in first frozen embryo transfer)	Higher diversity in the RIF group	Positive correlation with pregnancy rate	Significant differences are found between RIF patients and those who were pregnant in first frozen embryo cycle
Kong et al./2020, *Front. Med.* [102]	Vaginal	Total patients (*n*=475)	IVF Pregnancy (*n*=238) Non-pregnant (*n*=237)	Increased risk of IVF failure	Higher abundance of *Lactobacillus* in pregnancy group	Age, endometrial thickness, reduction in *Lactobacillus* and overgrowth of *Gardnerella*, *Atopbium* and *Prevotella* was strongly connected with IVF success

### Preterm birth

A strong body of evidence exists linking the risk of preterm birth and vaginal microbial composition, which has been reviewed in detail elsewhere [30,31]. The broad themes which emerge from these studies is that in many patient populations *Lactobacillus* spp. depletion is linked to the risk of both spontaneous preterm birth and preterm pre-labour rupture of membranes (PPROM). Several studies have shown that *L. crispatus* appears to be protective. There are also some data, largely in white Anglo-Saxon populations that *L. iners* is also a risk factor for both cervical shortening and for preterm birth. Our group has characterised the vaginal microbial communities longitudinally from 6-week-gestation and shown that dominance of the vaginal niche by non-*Lactobacillus* spp. associated with PPROM at all gestational age time points [32], highlighting that the early pregnancy microbiome can influence outcomes that occur at a later timepoint. Women who deliver at term are more likely to have a vaginal microbiota dominant in *L. crispatus* and patients who deliver preterm consistently show increased richness and diversity within the vaginal communities [33–36]. A prospective study examining the vaginal microbiota from patients with a dilated cervix pre- and post-rescue cerclage, identified reduced *Lactobacillus* spp. abundance in patients with premature cervical dilation and that that *G. vaginalis* was associated with unsuccessful rescue cerclage [37]. A recent study analysing the vaginal samples of 90 pregnancies that delivered at term and 45 spontaneous preterm birth patients, confirmed that those who delivered at term had a predominance in *L. crispatus*. The present study also found specific taxa were more abundant in preterm birth including BV associated bacterium 1 (BVAB-1), *Prevotella* species and *Sneathia amnii.* Metagenomic and metatranscriptomics analysis showed that expression of genes linked to the taxa identified by 16S rRNA and encoding for bacterial secretion systems, key in pathogenicity, was higher in the preterm birth cohort [38]. Table 2 gives examples of studies which have explored the relationship between the vaginal microbiota and risk of preterm birth.

**Table 2:** Examples of original research articles that explore the vaginal microbiota in relation to preterm birth

Author/year	Sample	Sample size	Population	Risk of adverse outcome		Findings
				High diversity	*L. crispatus/L. dominance*	
Preterm birth						
Hyman et al./2013, *Reprod. Sci.* [103]	Vaginal swabs	Term (*n*=66) Preterm birth (*n*=17)	Low and high risk for preterm birth	Increased risk in White patients		Higher *Lactobacillus* spp. content in low versus high risk for preterm birth
Romero et al./2014, Microbiome [11]	Vaginal swabs	Term control (*n*=72) Spontaneous preterm birth <34 weeks (*n*=18)	n/a	No	No	Increased relative abundance of *Lactobacillus* spp. as pregnancy progressed No difference in bacterial taxa between those who delivered at term or had preterm birth
Petricevic et al./2014, *Sci. Rep.* [104]	Vaginal swabs	Term (*n*=98) Preterm (*n*=13)	Low risk pregnant women sampled in early pregnancy		Decreased risk of preterm birth	*L.iners* as a single *Lactobacillus* spp. in early pregnancy may be involved in preterm birth
DiGuilio et al./2015, *Proc. Natl. Acad. Sci. U.S.A.* [105]	Vaginal swabs	Term (*n*=34) Preterm (*n*=15)	Low risk for preterm birth	Increased risk for preterm birth		Risk for preterm birth was higher in those with CST 4 followed by raised *Gardnerella* and *Ureaplasma* abundance
Nelson et al./2016, *Am. J. Perinatol.* [106]	Vaginal swabs	Term (*n*=27) Preterm (*n*=13)	Nulliparous Black women	No		The Shannon diversity index was not significantly different between the groups
Kindinger et al./2017, *Microbiome* [33]	Vaginal swabs	Term (*n*=127) Preterm birth <34 weeks (*n*=18) Preterm birth 34–37 weeks (*n*=16)	High risk	No	*L. crispatus* shown to be protective against preterm birth	*L. iners* dominance at 16 weeks is a risk factor for preterm birth (<34weeks). Cervical shortening and preterm birth were not associated with vaginal dysbiosis.
Stout et al./2017, *Am. J. Obstet. Gynaecol.* [35]	Vaginal swabs	Term (*n*=53) Preterm (*n*=24)	Mixed risk for preterm birth Predominately Black women. Preterm cohort included PTL, PROM and pre-eclampsia	Decrease in diversity in preterm birth group		Preterm birth is associated with increased vaginal microbiome instability compared with term. No distinct bacterial taxa correlated with preterm birth
Callahan et al./2017, *Proc. Natl. Acad. Sci. U.S.A.* [107]	Vaginal swabs	Term (*n*=85) Mainly White (*n*=30) Black (*n*=55) Preterm (*n*=50) Mainly White (*n*=9) Black (*n*=41)	White low risk and Black at high risk of preterm birth	Increased diversity in preterm birth within the predominately White cohort	*L. crispatus* low risk of preterm birth in both cohorts	Co-occurrence between *L. iners* and *Gardnerella.* No co-occurrence between *L. crispatus* and *Gardnerella*
Frietas et al./2018, *Microbiome* [10]	Vaginal swabs	Term (*n*=170) Spontaneous preterm birth (<37 weeks) (*n*=46)	Mixed risk cohort	Higher risk of preterm birth in those with increased diversity		No community structure predicted spontaneous preterm birth but there was an increase in diversity and *Mollicutes* prevalence
Brown et al./2018, *BMC Med.* [50]	Vaginal swabs	Term (*n*=20) PPROM (*n*=102) Before PPROM (*n*=15) After PPROM (*n*=87)	High risk (recruited from prematurity surveillance clinic)	Increased risk of PPROM and exacerbated by erythromycin treatment		*Lactobacillus* spp. depletion and *Sneathia* spp. were associated with early-onset neonatal sepsis
Tabatabaei et al/2019 BJOG [36]	Vaginal swabs	Term (*n*=356) Spontaneous preterm birth (*n*=94) <34 weeks (*n*=17) 34–36 weeks (*n*=77)	Low risk preterm birth	Increased risk of early (<34 weeks) but not late (34–36 weeks) preterm birth	Decreased risk of early (<34 weeks) but not late (34–36 weeks) preterm birth	Vaginal *Lactobacillus* and *Bifidobacterium* may lower the risk of preterm birth
Brown et al./2019, *Transl. Res.* [32]	Vaginal swabs	Term (*n*=36) PPROM (*n*=60)	Pregnant women high (*n*=38) and low (*n*=22) risk of preterm birth	Increased risk of PPROM	Decreased risk of PPROM	Increased instability of bacterial communities in PPROM patients in second trimester (increased *Prevotella*, *Streptococcus*, *Peptoniphilus* and *Dialister*
Elovitz et al./2019, *Nat. Commun.* [108]	Vaginal swabs	Term (*n*=432) Spontaneous preterm birth (*n*=107)	Mixed risk cohort for preterm birth	Increased risk of preterm birth in White women	Protective role in all subjects	Certain bacterial taxa were significantly associated with increased spontaneous preterm birth; *L. iners* and *A. vaginae* in white women and *M. curtsii/mulieris* and *Mageebacillus indolicus* in Black women
Brown et al./2019, *BJOG* [37]	Vaginal swabs	Term (*n*=30) Exposed foetal membranes (*n*=20)	High-risk patients undergoing rescue cerclage	Increased risk of cervical dilation and exposed membranes	Reduced risk	*G. vaginalis* prior to rescue cerclage was associated with cerclage failure
Fettweis et al./2019, *Nat. Med.* [38]	Vaginal swabs	Term (*n*=90) Preterm (*n*=45)	Predominately African ancestry (term and preterm cohort)	Increased risk of preterm birth	Decreased risk of preterm birth	Women who delivered preterm had higher levels of BVAB-1, *Sneathia amnii* and a group of *Prevotella* spp.
Payne et al./2021, *Am. J. Obstet. Gynaecol.* [109]	Vaginal swabs	Term (*n*=818) Spontaneous preterm birth (*n*=58) Non-spontaneous preterm birth (*n*=60)	Mainly White women	Increased risk of spontaneous preterm birth	Decreased risk of preterm birth	*G. vaginalis*, *L. iners* and *U. parvum* were strongly predictive of spontaneous preterm birth

Abbreviation: PTL, preterm labour.

#### The relationship between preterm birth and neonatal microbiota

The neonatal gut microbiota plays a crucial role in early life, especially in the maturation of the immune system and the metabolism of nutrients. *Bifidobacterium* is a key player in the neonatal gut microbiota, particularly present in vaginally delivered and breastfed infants. *Bifidobacterium* selectively digest sugars in breast milk and amino acids into lactic acid which helps to improve infant gut integrity [39]. The mode of delivery has been shown to affect the neonatal intestinal colonisation. Infants born vaginally acquire microbial communities that are related to the maternal gut and vagina. However, those born via caesarean section are mainly colonised by environmental bacteria and have lower numbers of Bifidobacteria and Bacteroides leading to lower diversity and impaired immune responses [40–42]. However, during the first year of life diet influences the gut microbiota and increases diversity [43]. Many studies have also shown that intestinal microbiota of preterm infants show less bacterial diversity, especially in the context of necrotising enterocolitis and late-onset sepsis, and differ considerably from the healthy term infant [44,45]. A longitudinal study collecting faecal samples in breast-fed extreme and moderately/very preterm infants (median gestational age: 26 and 30 weeks respectively) found that gestational age was the main driver of microbiota development. The predominance of *Enterococcus* was seen in the extremely premature infants but a transition to *Bifidobacterium* dominance occurred with increasing gestational age in both groups despite the mode of delivery. Antibiotics caused temporary changes in the microbial composition but there was recovery within 2–3 weeks. [46].

Group B *Streptococcus* (GBS) still remains one of the most common causes of neonatal sepsis. The most predictive factor is the presence of GBS in the maternal genital tract during childbirth [47]. A study investigating the relationship between GBS and the vaginal microbial composition in 428 non-pregnant patients found no correlation between CSTs and GBS status. However, specific taxa such as *Streptococcus* spp., *Prevotella biva* and *Veillonella* spp. were associated with GBS colonisation [48]. A study exploring the neonatal gut microbiota in GBS-positive women found enrichment of Enterococcaceae, Clostridiaceae and Ruminococcoceae in the infant gut at 6 months. However, long-term follow-up will be required to see whether these differences lead to adult disease later in life [49]. A prospective study examining the vaginal composition before and after PPROM correlated the findings to early-onset neonatal sepsis (EONS). The vaginal microbiota prior to delivery in those cases of placental chorioamnionitis and funisitis found an enrichment of *Prevotella, Sneathia, Peptostreptococcus* and *Catonella* spp. and reduced *Lactobacillus* spp. compared with patients with normal histology. In the cases with EONS, the maternal vaginal microbiota prior to delivery was enriched with *Catonella* spp *and Sneathia spp.* whereas *L. crispatus* was overrepresented in those who did not develop EONS [50]. This highlights the key involvement of the vaginal microbiota in the development of preterm neonatal sepsis and the potential role for modifying the composition to positively influence neonatal outcome.

## Placental microbiota

Much recent research attention has been directed toward establishing whether there is a physiologically normal and functional placental microbiome, abnormalities or imbalances of which may contribute to adverse pregnancy outcome. The belief that reproduction occurs in a sterile environment was supported by studies using culture-based techniques, which failed to detect bacteria in the placenta of healthy pregnancies [51]. However, using highly sensitive bacterial DNA sequencing approaches, Aagaard et al. published the first study that proposed a unique non-pathogenic placental microbiota niche [52] and that the placental microbiota that differed between term and preterm deliveries [53–55]. While it was originally suggested that that these organisms contribute to metabolic function, their low biomass in the placenta questioned the physiological significance. Organisms reported to contribute to the placental microbiota included those commonly found in soda lakes and marine environments [30] suggesting that this apparent placental microbiome is actually reagent and laboratory contamination and that apparent differences, for example between term and preterm cases, were due to batch effects [56]. Recent studies have addressed this issue by extensively matching the cases being investigated with laboratory controls and could not identify a microbiota within the placenta that was distinguishable from background technical controls [57,58]. Even in studies where distinct bacteria have been detected by molecular techniques, it is unclear whether these are viable organisms or dead material. The placenta has a role in removing organisms and there is a risk such sensitive techniques are amplifying these cleared microbes. The anatomical, physiological and immunological barriers that exist at the maternal–foetal interface to prevent microbial invasion also argue against the likelihood of a normal placental microbiome. If a unique microbiome existed in the placenta an immunologically naïve foetus could be overwhelmed [51]. Therefore, there may be bacteria present at a low level in the placenta but given the function of the placenta, normal bacterial colonisation and development of a placental niche seems unlikely [59].

A recent study by Goffau et al. [60] found no evidence of bacterial signals from placental samples of pregnancies complicated by preterm birth, pre-eclampsia and small for gestational age infants (*n*=318) or uncomplicated pregnancies (*n*=219). This study found that the main source of bacterial DNA was from the laboratory reagents. However, *Streptococcus agalactiae* (GBS) was detected using 16S rRNA amplification and verified by metagenomics and qPCR, in the placenta of 5% of women prior to the onset of labour, although there was no association with complicated pregnancies. Therefore, while the study concluded that a resident placental microbiome did not exist, bacterial placental colonisation can be present although is unlikely to contribute to the majority of complicated pregnancies. Studies that have examined pathogens such as *Salmonella* on human placental villous explants from different gestational ages demonstrated that the bacterial burden was highest in the first trimester explants. Therefore, the first trimester may be a more vulnerable time and placental colonisation infection needs to be carefully considered in relation to poor outcomes at this gestation [61]. In the case of spontaneous preterm birth associated with chorioamnionitis-specific bacteria such as *Mycoplasma* spp. and *Ureaplasma* spp. have been identified in the placenta [62,63]. Therefore, while the evidence to support a functional placental microbiota is weak, in some cases there may be placental pathogenic colonisation seeded from ascending vaginal infection or haematogenous spread. It is also notable that the majority of studies investigating evidence of placental microbial colonisation have focused on term and preterm placentas collected vaginally or by caesarean section. Table 3 summarises the current evidence regarding the placental microbiome in pregnancy at any gestational age.

**Table 3:** Examples of current evidence to date investigating the human placental microbiota at any gestation

Author/year	Sample	Sample size	Mode of delivery	Techniques	Contamination Control	Findings
Aagaard et al./2014/Science translational medicine [52]	Villous tree	Term (*n*=231) Preterm (*n*=89)	Term Caesarean (*n*=53) Term Vaginal (*n*=178) Preterm Caesarean (*n*=20) Preterm Vaginal (*n*=69)	16S rRNA gene sequencing V1-3 Metagenomics (subset *n*=48)	1 blank extraction kit per 11 placental samples (no bands routinely amplified) Reagents were sequenced when non-human sources were identified but details not provided	There is a unique low abundance placental microbiome. There are observed similarities in non-pregnant oral and placental microbiomes. The placental microbiome differs between preterm and term women and in those with and without antenatal infection
Doyle et al./2014/Placenta [54]	Placental membranes (chorion and amnion)	Spontaneous preterm birth (*n*=14) Term (*n*=10)	Preterm Vaginal (*n*=14) Term Caesarean (*n*=4) Term Vaginal (*n*=6)	16S rRNA gene sequencing V1-2 and V5-7	No	Bacterial DNA present in preterm and term placental membranes irrespective of mode of delivery A consistently identifiable bacterial species in preterm labour
Antony et al./2015/Am J Obstet Gynecol. [55]	1 × 1 × 1 cm cuboidal section excised from different areas of placenta	Term (*n*=175) Preterm (*n=*62)	Caesarean (*n*=54) Vaginal (*n*=183)	16S rRNA gene sequencing V1-3	No	Excess gestational weight gain associated with altered placental microbiome and metabolic profile in preterm birth patients
Zheng et al./2015/Nutrients. [110]	Placenta 4 × 1 cm^3^ cuboidal sections (decidua and foetal chorion discarded)	Low birth weight <3 kg (*n*=12) Normal birth weight ≥3 kg to <4 kg (*n*=12)	Vaginal (*n*=24) Caesarean (*n*=0)	16S rRNA gene sequencing V3-4	No	There is a placental microbiome. The placentas of low birthweight neonates had lower bacterial richness and evenness compared with normal birthweight neonates
Bassols et al./2016/Pediatric research [111]	Villous tree	Gestational Diabetes (*n*=11) Without Gestational Diabetes (*n*=11)	Vaginal (*n*=22) Caesarean (*n*=0)	16S rRNA gene sequencing V3-4	No	A distinct microbiota profile is present in the placental samples of patients with gestational diabetes
Collado et al./2016/Scientific reports [53]	Placenta Amniotic fluid Colostrum Meconium	Infant mother pairs (*n*=15)	Term Caesarean (*n*=15)	16S rRNA gene sequencing V1-3 Anaerobic culture of placenta and amniotic fluid samples	No	Placenta and amniotic fluid harbour unique microbial communities. Meconium shares features with the microbiota in placenta, amniotic fluid and colostrum. Foetal intestinal colonisation could be initiated *in utero*. *Staphylococcus* and *Propionibacterium* were cultured from placenta
Lauder et al./2016/Microbiome. [57]	Placenta (basal plate biopsy and foetal side biopsy)	Term (*n*=6)	Caesarean (*n*=1) Vaginal delivery (*n*=5)	16S rRNA sequencing V1-2 qPCR	Laboratory air swabs (*n*=11) Sterile swabs (*n*=8) Blank extraction kits (*n*=8)	Microbial signatures in placental tissue could not be distinguished from technical controls
Prince et al./2016/Am J Obstet Gynecol [63]	Swabs from chorion or villous membrane adjacent to foetal side	Term (*n*=27) Preterm (*n*=44) Term Chorioamnionitis (*n*=12) Preterm Chorioamnionitis Mild (*n*=11) Severe (*n*=20)	Term Cesarean (*n*=7) Term Vaginal (*n*=20) Preterm Caesarean (*n*=7) Preterm vaginal (*n*=37)	Metagenomics Culture for *Ureaplasma* or *Mycoplasma spp.*	No Only yields with high reads were included in analysis without concern for contamination	Spontaneous preterm birth patients have a placental microbiota that differed by severity of chorioamnionitis
Doyle et al./2017/Placenta. [112]	Amnion and Chorion	1097 subjects *Rural Malawi setting	Unreported vaginal, caesarean, preterm and chorioamnionitis cases	16S rRNA gene sequencing V5-7 qPCR	Reagents from blank extraction kit sequenced for every ten extractions. Only placental samples that were positive for bacterial DNA (defined as 40 CFU/μl) were sequenced. Sample processing delays increased the chance of positive qPCR	A distinct placental microbiome exists. 68.1% of amnion–chorion and 46.8% placental samples had positive qPCR. A varied placental microbial structure is associated with severe chorioamnionitis. The source of bacteria in the placenta overlapped with the vagina and not the oral cavity
Gomez-Arango et al./2017/Scientific reports [113]	Placental biopsy from foetal side. Matched oral and faecal samples	37 patients Overweight (*n*=13) Obese (*n*=24)	Term Caesarean (*n*=17) Term Vaginal (*n*=20)	16S rRNA gene sequencing V6-8	Reagent, DNA extraction and PCR control pooled and sequenced for each kit type. Any OTUs detected were removed from analysis	A placental microbiome was identified irrespective of mode of delivery. Placental communities shared more similarity to oral microbiome than gut but this declined with each taxonomic level
Parnell et al./2017/Scientific reports. [114]	Placenta: Basal plate Villous tree Foetal membrane	57 Term Women	Term Cesarean (*n*=34) Term Vaginal (*n*=23)	16S rRNA gene sequencing V1-9 (V7/8 did not amplify.V1,5,9 amplified less than half and V2 showed environmental contaminants) qPCR conducted on V4 region	Water Control *n*=5 and Regent test blanks *n*=8 Negative controls occasionally had 34 copies/μl. Only positive qPCR in placental samples were included (if >34 copies/μl)	Tissue-specific profiles identified in placental microbiome. Variation is seen in the placental microbiota between amnion–chorion and basal plate.
Zheng et al./2017/Oncotarget [115]	Placenta 4 × 1 cm^3^ cuboidal sections (decidua and foetal chorion discarded)	Term without macrosomia (*n*=10) Macrosomia birth weight > 4 kg (*n*=10)	Caesarean (*n*=20)	16S rRNA gene sequencing V3-4	No	Distinct placental microbiota profile in foetal macrosomia
Leon et al./2018/Appl Environ Microbiol [62]	Placental Villous tree	256 patients Term (*n*=165) Preterm (*n*=91)	Caesarean Term (*n*=81) Vaginal Term (*n*=84) Caesarean Preterm (*n*=55) Vaginal Preterm (*n*=36)	16S rRNA gene sequencing V5-7	Negative extractions and PCR blanks were examined. Samples ≥ 500 reads were analysed (*n*=19)	Low level relatively diverse placental microbial signature is present in normal and complicated pregnancies. There was overlap between technical controls and placental samples A unique preterm placenta did not exist but *Ureaplasma* and *Mycoplasma* enriched the spontaneous preterm birth cohort.
Seferovic et al./2019/Am J Obstet Gynecol [116]	Placental villous tree	53 patients Term (*n*=26) Preterm (*n*=26) One positive control with histological chorioamnionitis	Term Caesarean (*n*=22) Term Vaginal (*n*=4) Preterm Caesarean (*n*=8) Preterm Vaginal (*n*=18)	*In situ* hybridisation against conserved region of 16 S ribosome. 16S rRNA sequencing V4 Metagenomics	Environmental swab cultures (inside and outside placental containers). Kit-negative extractions, *n*=6	Very low biomass bacteria were observed by histological and 16S rRNA gene sequencing distinct from environmental controls. Unclear if commensal microbial abundance varies in preterm and term pregnancies. Viability of organisms unknown
De Goffau et al./2019/Nature [60]	Placental terminal villi	537 women Adverse pregnancy outcome (*n*=318) Controls (*n*=219)	Caesarean SGA (*n*=20) PET (*n*=20) Control (*n*=40) Vaginal and Caesarean SGA (*n*=100) PET (*n*=100) Control (*n*=198) Preterm (*n*=100)	16S rRNA V1-2 Metagenomics qPCR for *S. agalactiae*	Positive control using *S. bongori* to compare 16S rRNA with metagenomics For each DNA isolation kit extraction blanks were carried out	No evidence to support a placental microbiome. No relationship between placental infection and SGA, PET or preterm birth. The major source of bacterial DNA in the samples was contamination from laboratory reagents. The only organism consistently present in the placenta of 5% of women prior to labour (detected by three methods) was *S. agalactiae*
Theis et al./2019/Am J Obstet Gynecol [58]	Amnion–chorion plate Villous tree	Healthy Term women (*n*=29)	Term Caesarean (*n*=29)	16S rRNA V4 qPCR Metagenomic surveys Bacterial culture	DNA extraction kits (*n*=6) Laboratory environmental controls (*n*=16) Operating rooms (*n*=21)	No consistent evidence the placenta harbours a unique microbiota. 28/29 placental samples did not yield bacterial cultivars 18 prominent OTUs accounted for 90% of placental tissue and 86.4% of background technical controls There were no consistent differences in the composition of placental samples and technical controls
Gschwind et al./2020/PLoS One [117]	Chorionic villi Umbilical cords Foetal membranes	Healthy Term pregnancy (*n*=38)	Caesarean (*n*=29) Vaginal (*n*=9)	16S rRNA V8-9 qPCR V4 Bacterial culture Metagenomics	16 Extraction blanks (*n*=16) Reagent extraction kit controls (*n*=3) Culture media and incubation condition controls (*n*=38)	Placenta does not harbour specific consistent functional microbiota No significant viable bacteria or bacterial DNA in the *in utero* samples collected from caesarean section
Sterpu et al./2020/Am J Obstet Gynecol [59]	Placenta (maternal, middle and foetal side) Saliva Vaginal Rectal Amniotic fluid Vernix	76 Term pregnancies	Term Caesarean (*n*=50) Term Vaginal (*n*=26)	Metagenomics qPCR 16S rRNA V6-8 Bacterial culture	PCR reagents DNA extraction controls	16S rRNA gene sequencing and qPCR found bacterial signals that were not distinguishable from background controls No meaningful comparisons could be made to oral, faecal or vaginal samples Very few genera detected by16S rRNA sequencing could be confirmed by culture
Olomu et al./2020/BMC Microbiol. [118]	Parenchymal placental tissue Vaginal Rectal Maternal blood Cord blood	Term patients (*n*=47) GDM (*n*=16) Obese (*n*=16) Normal weight (*n*=15)	Term Caesarean (*n*=47)	16S rRNA V3-4 qPCR	Multiple negative or blank controls. Sterile swabs exposed to operating rooms or air in sampling room. Reagent, Kit and sequencing reaction controls	No distinct microbiome existed in placental samples that differed from blank controls An additional source of cross contamination was identified from high biomass samples being analysed adjacent to low biomass samples
Oliveira et al./2020/Epidemiol Infect [29]	Endocervical swabs Placental tissue	Miscarriage patients (*n*=89) Control with no history of miscarriage (*n*=20)	Miscarriage patients undergoing curettage 8–20 weeks gestation (*n*=89) Term pregnancies vaginal deliveries (*n*=20)	qPCR to detect *M. genitalium, M. hominis, U. parvum, U. urealyticum* and *N. gonorrhoeae*	No	Women with *Mollicutes* detected in placenta had a seven-fold higher chance of miscarriage. A positive association between *U. parvum* in placental tissue and miscarriage

Abbreviations: CFU, colony forming unit; GDM, gestational diabetes mellitis; OTU, operational taxonomic unit; PET: pre-eclampsia; SGA, small for gestational age.

## Endometrial microbiome in reproduction

An increasing body of work has focused on the existence of the endometrial microbiome. While many studies are confounded by vaginal contamination and low biomass there is emerging evidence that the lower uterine microbiome is distinct and could be contributed to by the vaginal microbiota [64]. A systematic review assessing the effect of the endometrial microbiome on artificial reproductive technologies (ARTs) showed that an abnormal endometrial microbiome was associated with poor ART outcomes [65]. More recent work has described the use of molecular approaches to characterise the endometrial microbiota at the time of single euploid embryo transfer which amplified bacteria within the embryo catheter tip [66]. A study evaluating paired endometrial fluid and vaginal aspirates samples in 13 women found different bacterial genera in the uterine cavity compared with paired vaginal samples. The presence of non-*Lactobacillus* dominated microbiota in the endometrium was also associated with decreased implantation and live birth rates [67]. These findings were corroborated in slightly larger studies where a non-*Lactobacillus* dominated endometrial microbiota was higher in infertile patients [68]. Nonetheless small sample sizes and limited laboratory contamination controls have not been able to address the impact of cross-contamination from the high biomass in the vagina. The relatively low biomass in the uterine cavity can also lead to molecular techniques being susceptible to background contamination. Studies have tried to account for these limitations by collecting samples from abdominal hysterectomies and incorporating extraction kit controls. Winters et al., reported a resident microbiota in the middle endometrium of 60% of women undergoing a hysterectomy that principally consisted of *Acinetobacte*r, *Pseudomonas*, Comamonadaceae and *Cloacibacterium* that were not present in background technical controls, or other body sites except the cervix [69]. Studies that collect samples abdominally also corroborate these microbial profiles and rarely detect high levels of *Lactobacillus* spp. within the endometrium [64]. Nonetheless these results need to be verified by complementary techniques such as microscopy and culture and further studies are required to understand the signalling pathways activated by these microbes and the metabolites synthesised to appreciate the impact on reproduction and fertility [70]. Table 4 demonstrates the current studies to date that have evaluated the endometrial microbiota.

**Table 4:** Examples of current evidence exploring the endometrial microbiota in reproduction

Author/year	Sampling	Sample size	Population	Techniques	Contamination controls	Findings
Kyono et al./2019, *Reprod. Med. Biol.* [119]	Endometrial fluid samples collected using IUI catheter	92 women	IVF	Endometrial flora test (Varinos Inc.)	No	Pregnancy rates were not significantly different between *Lactobacillus* dominant and non-dominant groups
Grau et al./2019 *Pathogens* [120]	Endometrial fluid (six samples from one patient)	Case report	Infertile patient with history of ectopic pregnancy and two miscarriages	16S rRNA sequencing and whole metagenome sequencing	No	This patient had persistent endometrial *G. vaginalis* colonisation and virulence genes involved in biofilm and antibiotic resistance
Liu et al./2019 *Fertil. Steril.* [121]	Endometrial biopsy and fluid (7 days after LH surge)	130 infertile women	Infertile women with chronic endometritis (*n*=12) and without (*n*=118)	16S rRNA sequencing V4	16 negative controls (8 air swabs and 8 collection controls) Extremely low sequence reads	Bacteria such as *Dialister*, *Bifidobacterium, Prevotella Gardnerella* and *Anaerococcus* are more abundant in the endometrial microbiota of women with CE than those without *Lactobacillus* spp. was more abundant in non-CE microbiota
Kyono et al./2018, *Reprod. Med. Biol.* [68]	Endometrial fluid and vaginal samples	102 women	IVF (*n*=79) Non-IVF (*n*=23) Healthy controls (*n*=7)	16S rRNA sequencing V4	No	Increased *Lactobacillus* spp. in endometrium of healthy volunteers compared with infertility patients
Hashimoto et al./2019, *J. Assist. Reprod. Genet.* [122]	Endometrial fluid	99 women	IVF	16S rRNA sequencing V4	Yes-blank extraction controls and known regent contaminants such as *Sphingomonas* and *Stenotrophomonas* were excluded	No difference in pregnancy or miscarriage rate between eubiotic or dysbiotic endometrium
Winters et al./2019, *Sci. Rep.* [69]	Mid endometrial Rectal Vaginal samples	25 women	Patients having a hysterectomy primarily for fibroids	16S rRNA sequencing V1-2 and qPCR	Background DNA controls	60% of the mid endometrial samples had a bacterial load that exceeded background controls and was distinct from other body sites
Chen et al./2017, *Nat. Commun.* [64]	Endometrial Vagina Cervical mucus Peritoneal fluid Fallopian tubes	95 women having surgery for non-infectious conditions	Samples from peritoneal and uterine sites were taken during abdominal surgery	16S rRNA sequencing V4-5 qPCR	Negative controls (sterile swabs from surgeons gloves, and patients skin) Negative laboratory controls	Distinct communities were identified in uterus, fallopian tubes, peritoneal fluid that differed from the vagina
Kitaya et al./2019, *Mediators Inflamm.* [123]	Endometrial fluid and vaginal samples	46 women	History of RIF (*n*=28) Infertile women undergoing first IVF (*n*=18)	16S rRNA sequencing V4	Blank water controls Known contaminants were excluded from endometrial samples	Endometrial microbiota showed significant variation between RIF and control group
Carosso et al./2020, *J Assist. Reprod. Genet.* [124]	Vaginal Swab Embryo catheter tip	15 women	Does ovarian stimulation and progesterone supplementation modify the microbiota in women having IVF	16S rRNA sequencing V3-4-6	Blank extraction kit controls *Sphingomonas* excluded from analysis as known contaminant from previous work	Endometrial microbiota was heterogeneous Endometrial *Lactobacillus* spp. was reduced following controlled ovarian stimulation and progesterone supplements

Abbreviation: CE, chronic endometritis.

## Mechanisms that link the vaginal microbiota to pregnancy outcomes

Many of the studies exploring the link between the vaginal microbiota and pregnancy outcomes have been associated with little insight into the mechanisms that trigger adverse events. Nonetheless, the protective effects of *Lactobacillus* species against pathogen colonisation are quite well described. Lactobacilli utilise breakdown products of glycogen within the vagina to produce lactic acid which creates an acidic pH that deters the growth of many other bacteria, as well as up-regulating autophagy which clears intracellular pathogens from vaginal epithelial cells [71]. Lactobacilli also produce bacteriocins to eliminate other bacteria and strengthen their dominance [72,73]. *L. crispatus*, *L. gasseri* and *L. jensenii* produce both the l and d-isomers of lactic acid whereas *L. iners* has a smaller genome that lacks the gene encoding enzyme required to produce d-lactic acid. Relevant to reproductive tract health, the d-isomer of lactic acid has been shown to down-regulate matrix metalloproteinase-8 (MMP-8) production which breaks down the cervical plug that inhibits entry of bacteria to the upper genital tract [71]. Moreover, vaginal microbiota dominant in *L. crispatus* demonstrate increased autophagy and lower cellular stress compared with women dominated by *L. iners* [74]. Therefore even within the *Lactobacillus* genera, certain species such as *L. iners* are not as effective at out competing other species and thus associated with transitions to ‘high diversity’ microbial states [75]. A recent study investigating the interaction between the different strains of Lactobacilli and decidualised endometrial cells *in vitro* found that *L. crispatus* was significantly more successful at attaching to the host cells compared with other *Lactobacillus* strains. In addition the interaction between *Lactobacillus* and endometrial cells did not cause inflammation or host cell death [76].

Many studies have focused on the correlation between high-diversity vaginal microbial composition and inflammatory mediators as an explanation for poor outcomes. Kindinger et al. [77] reported in a case–control study of nearly 700 patients that pregnancy outcome in women at high risk of preterm birth who had undergone cervical cerclage was highly dependent upon the suture material used for the procedure. Use of the commonly used braided suture material, compared with monofilament material, was associated with increased risk of both intrauterine foetal death and preterm birth. The braided material was shown to induce, in some women, a persistent shift towards a reduced *Lactobacillus* spp. composition and enrichment of pathobionts. This was associated with increased inflammatory cytokines and interstitial collagenase excretion into the cervicovaginal fluid and early remodelling of the cervix. This uncovered how the interaction with the host could induce an adverse microbial composition and therefore alter reproductive outcomes. Other longitudinal cohort studies have also demonstrated that preterm birth associated taxa correlate with pro inflammatory cytokines in the cervicovaginal fluid [38], although this is influenced by host ethnicity and probably genetics and the ultimate adverse outcome involves the interplay between the microbiota and immune system.

## Modifying the cervicovaginal microbiota

### Antibiotics

The current international guidance for the treatment of vaginal conditions such as BV recommends metronidazole, clindamycin or tinidazole administered orally or vaginally. However, high recurrence rates are still reported following treatment and many studies report antimicrobial resistance [78]. Antibiotics themselves may be directly harmful in early pregnancy and can increase the risk of spontaneous miscarriage. Macrolides, quinolones and tetracyclines all increased the risk of miscarriage and should be given with caution [79]. However, the large body of evidence that vaginal microbial composition can influence reproductive outcomes, suggests the possible therapeutic role of agents that can change that composition. Several have examined the role of antibiotics in pregnant patients with BV in relation to the risk of preterm birth. The largest randomised control trial, which screened 84530 women in early pregnancy and randomised 3105 women with BV to an oral clindamycin treatment arm and placebo arm, found no risk reduction for late miscarriage (16–22 weeks) or spontaneous very preterm birth (22–32 weeks) [80]. A subsequent metanalysis confirmed these findings [81] although highlighted heterogeneity of the studies included, with different patient cohorts and antibiotic regimes being compared. Antimicrobial resistance genes are present in the vaginal microbiome of patients with BV symptoms may also influence the use of antibiotics in this field [82]. The formation of biofilms are now implicated in BV and the inability of antimicrobials to penetrate this matrix is also likely to result in treatment failure and resistance [83].

There is currently no evidence that antibiotic prophylaxis reduces the risk of preterm birth [84]. The ORACLE-II Study showed that, in women in spontaneous preterm labour, neither erythromycin, co-amoxiclav or a combination of the two had any effect upon a composite outcome of neonatal death, chronic lung disease, or major cerebral abnormality on ultrasonography before discharge from hospital [85]. Its follow-up study showed that the prescription of antibiotics was associated with an increase in functional impairment among their children at 7 years of age although the overall risk was low. The ORACLE-I study, in women with PPROM showed that the composite outcome of short-term respiratory function, chronic lung disease and major neonatal cerebral abnormality was reduced with the use of erythromycin. But the use of co-amoxiclav was associated with a significant increase in the occurrence of neonatal-necrotising enterocolitis [86]. At the 7-year follow-up neither antibiotic had a significant effect on the overall level of behavioural difficulties experienced, on specific medical conditions or on the proportions of children achieving each level in reading, writing or mathematics at key stage one [87]. A recent study has shown that prescription of erythromycin in after PPROM has a tendency to convert a healthy *Lactobacillus*-dominant vagina microbiota into *Lactobacillus* depletion which is then a risk factor for early adverse neonatal outcomes [50]. It is likely that for antibiotics to have any benefit in these contexts; we will need to develop tools to allow them to be properly targeted at well-phenotyped individuals.

### Live biotherapeutics: probiotics

There is a growing interest in the potential to modulate the vaginal microbiota using probiotics or live biotherapeutic products. A systematic review of oral probiotic use in pregnant women at low risk for preterm birth did not find a reduction in the incidence of preterm birth (<37 weeks) [88]. Recent studies have shown that oral probiotics administered in early pregnancy do not modify the vaginal microbiota [89,90]. Subsequently, a systematic review evaluated the use of vaginal probiotics in BV and vulvovaginal candidiasis. The use of vaginal probiotics was promising in BV cure and prevention, but of the 13 studies included, 5 had medium and 8 had high overall risk of bias. There was also minimal detection of probiotic strains after the dosing period, implying a lack of colonisation. There was considerable heterogeneity in these trials in terms of probiotic strain, length of use and duration between last probiotic use and vaginal sample collection [91]. It is probably the case that it will not be possible to colonise the vagina with live biotherapeutics administered orally. The apparent protective effect of *L. crispatus* in preterm birth, and perhaps also in miscarriage and other adverse pregnancy outcomes suggests that a live biotherapeutic containing that organism and administered vaginally, might be therapeutically valuable. A recent randomised double-blind placebo controlled trial in 228 women found vaginally administered *L. crispatus* prevented BV recurrence after metronidazole treatment [92]. This work encourages future trials to focus on vaginal administration of *L. crispatus* in pregnancy to influence the vaginal composition and improve pregnancy outcome.

### Vaginal microbiome transplant

Although the vaginal microbiota is much less rich and diverse than the microbiota of other body compartments, especially the gut, it nevertheless remains possible that most effective colonisation strategy would be achieved by biotherapeutic treatment using a community of organisms, rather than a single pure organism. The use of faecal microbiota transplantation has been successful in treating recurrent *Clostridium difficile* infection and this has led to subsequent interest in the use of transplanted human material to alter the vaginal microbial composition [93]. The first exploratory study that used vaginal microbiome transplantation (VMT) targeted five patients with recurrent and antibiotic non-responsive BV. In this pilot study, four of the five patients had long-term remission, which was defined as symptom improvement and microscopic vaginal fluid appearance of a *Lactobacillus-*dominated vaginal microbiome at 5–21 months after VMT. Recurrent VMT was needed in three patients but overall long lasting improvements were seen with a post-VMT compositional change dominated in *Lactobacillus* genus. These preliminary results call for randomised control trials to help understand the therapeutic efficacy in women with intractable BV. Given the small sample size the potential risks of this procedure cannot be discounted even though no adverse effects were reported [94].

As mentioned above, the mode of delivery is also thought to have an impact on the microbial composition in newborns and associations have been reported between caesarean section deliveries and an increased risk of obesity and asthma [95,96]. Although a causal link and mechanism has not been identified, reports suggest the altered microbial composition may impact development of the host immune system and metabolism [97]. A recent pilot study explored exposing newborns to maternal vaginal contents following a caesarean section and found the neonatal gut, oral and skin microbiome was enriched with vaginal bacteria similar to those infants born vaginally. Such organisms were often underrepresented in unexposed caesarean section infants. Nonetheless the sample size was limited, and sampling was only within the first month after birth [98]. Therefore, it is unclear whether such vaginal communities continue to persist in the infant or have any influence on future disease outcomes. Given the complex nature of labour and the lack of understanding between the host–microbe interactions and neonatal immune system, further research is required to evaluate the full potential of this process.

## Conclusion

There is a great deal of evidence that demonstrates the reproductive tract microbiota can influence pregnancy outcome. Nonetheless, a great deal needs to be uncovered with regard to the mechanisms that trigger adverse events and the relationship between microbial composition and the immune system. A recurring theme that populates the current literature is that *L. crispatus* is beneficial to the host and possess key properties that create a stable environment. This paves the way for therapeutic intervention that modifies the microbiome and provides exciting new developments for translational research.

## Open Access

Open access for this article was enabled by the participation of Imperial College London in an all-inclusive *Read & Publish* pilot with Portland Press and the Biochemical Society under a transformative agreement with JISC.

## Abbreviations

ART: artificial reproductive technology
BV: bacterial vaginosis
CST: community state type
EONS: early-onset neonatal sepsis
GBS: Group B *Streptococcus*
IVF: *in-vitro* fertilisation
PPROM: preterm pre-labour rupture of membrane
qPCR: quantitative polymerase chain reaction
RIF: recurrent implantation failure
VALENCIA: VAginaL community state typE Nearest Centroid clAssifier
VMT: vaginal microbiome transplantation
